# Caregivers’ Sensemaking of Children’s Hereditary Angioedema: A Semiotic Narrative Analysis of the Sense of Grip on the Disease

**DOI:** 10.3389/fpsyg.2019.02609

**Published:** 2019-11-27

**Authors:** Maria Francesca Freda, Livia Savarese, Pasquale Dolce, Raffaele De Luca Picione

**Affiliations:** ^1^Department of Humanistic Studies, University of Naples Federico II, Naples, Italy; ^2^Department of Public Health, University of Naples Federico II, Naples, Italy; ^3^Giustino Fortunato University, Benevento, Italy

**Keywords:** caregiver, chronic disease, illness narratives, sensemaking, hereditary angioedema

## Abstract

**Background and aims**: In pediatrics receiving a diagnosis of a chronic condition is a matter that involves caregivers at first. Beyond the basic issues of caring for the physical condition of the ill child, how caregivers face and make sense of the disease orients and co-constructs their children’s sensemaking processes of the disease itself. The aim of this article is to explore the experience of a rare chronic illness, a pediatric case of Hereditary Angioedema (HAE) from the caregivers’ perspective. Hereditary angioedema is characterized by subcutaneous swellings that can involve internal as well as external mucosal tissues and is highly variable and unpredictable in terms of severity, frequency, and where it occurs.

A qualitative narrative semiotic analysis of n. 28 maternal narratives on their children’s disease experience. Narratives were collected by an *ad hoc* interview on three domains of the disease experience: (A) interpretation of disease variability, (B) dialogical processes, and (C) management of the disease. Subsequently, we executed a TwoStep cluster analysis for categorical data to detect cross-sectional profiles of the maternal sensemaking processes of the disease.

**Results**: The coding grid was built analyzing the characteristics of the narrative links that orient the connection between the elements of the experience within each domain: (A) the connection among events, for the domain of disease variability interpretation, (B) the connection between self and other, for the dialogue domain, and (C) the connection among sensemaking and actions, for the disease management domain. Results from the cluster analysis show three narrative profiles: (1) adempitive; (2) reactive; (3) dynamic.

**Discussion**: Profiles will be discussed in light of the general conceptual framework of the Sense of Grip on the Disease (SoGoD) highlighting the importance of those sensemaking processes which, instead of relying on a coherent and closed interpretation of the disease, are characterized by a degree of tolerance for uncertainty and the unknown.

## Introduction

Living with a chronic disease is a critical condition that has become increasingly common in the last two decades. This is to address to new medical advancements which are leading to the identification of several genetic syndromes, as well as to increasingly effective therapies ensuring a good enough quality of life even in the case of previously incurable conditions (Graffigna et al., 2017; Robinson, 2017). Chronicity is therefore becoming a brand new clinical condition with which medicine and health professionals must contend (Tattersall, 2002; Thorne et al., 2002; Paterson, 2003; Venuleo et al., 2018). Sanitary settings and caregivers are called to respond to the physical and psychological needs of patients on a daily basis, for their entire life. This new condition requires a transformation of the Health Service as well as of the caregiving system in order to reduce the economic and human burden of chronicity (De Ridder et al., 2008; Quattropani et al., 2018a; Forestier et al., 2019). In this light, thanks to recent early detection techniques, the number of chronic conditions in pediatrics is increasing (Compas et al., 2012; Cipolletta et al., 2015). The Health Service is called upon to respond to life-span care needs using *ad hoc* comprehensive models of treatment and care (Perrin et al., 2007). In such cases, receiving a diagnosis of a chronic condition in pediatrics is a matter that involves parents as the primary caregivers at first (Cipolletta and Amicucci, 2015). In fact, the discovery that a child suffers from a chronic condition can impact parents’ life as if they were personally affected by the disease. In facing such a critical event, parents are expected to respond to various challenges – sometimes antithetical – while executing their role. They must attempt to elaborate their personal feelings of suffering for their children’s disease and, simultaneously, as caregivers, they must care for their children’s physical and emotional health, promoting their comprehension of the disease (Marvin and Pianta, 1996; Milshtein et al., 2009; Eccleston et al., 2015; Freda et al., 2016a).

Beyond the basic issues of caring for the physical condition of the ill child, how caregivers face and make sense of the disease orients and co-constructs their children’s sensemaking processes of the disease itself. In fact, the sensemaking exchanges between caregivers and young patients in these formative years of childhood and adolescence are fundamental for the development of a sense framework that can help children become competent and responsible adults in the psychological and physical adjustment to the disease (Lewandowski and Drotar, 2006; Armstrong et al., 2011; Distelberg et al., 2014; Parrello and Giacco, 2014; Manna and Boursier, 2018). Children’s permeability to caregivers’ stimuli is intrinsic to the developmental immaturity of their mind. Neuroscience is echoing developmental psychology in highlighting how variable sensemaking patterns and behaviors learned by interaction with significant others are (Tronick, 2010; Ginot, 2015). This awareness makes it highly relevant to focus on caregivers’ and, in particular, on the maternal processes of elaboration, since they can become active catalysts of virtuous cycles of sensemaking of the disease. In this study, we investigate the condition of living with a specific chronic rare disease, hereditary angioedema, from the caregivers’ perspective. We focus on the perspective of mothers since, within our context, they are still the parent which is most devoted to childcare.

Hereditary Angioedema (HAE) is characterized by subcutaneous swellings that can involve the mucosal tissues of the arms, legs, hands, and feet, while internal organ involvement can affect abdominal and laryngeal tissues. External swelling events frequently cause discomfort at a social and psychological level by deforming, even if temporarily, certain parts of the body. Swelling involving the abdominal or laryngeal tissues causes intense pain and is quite threatening to the health of the subject (Cicardi et al., 2014).

These swellings are called “attacks” in jargon and are highly variable and unpredictable in terms of localization, severity, and frequency, both from an inter-individual as well as an intra-individual perspective (Zuraw, 2008). Such attacks hinder the completion of daily activities such as going to school or working, meeting friends, or practicing sports and hobbies (Lumry et al., 2013). Despite the latest research leading to an ever-widening view of the disease’s genetic mutations and to the identification of effective drugs aimed at blocking and reducing the severity of the attacks, little is yet understood about the factors that trigger HAE attacks (Freda et al., 2016a). The lack of knowledge pertaining to HAE variability forces people affected by it and their caregivers to live with a constant sense of uncertainty and concern that an attack could occur at any time (Savarese et al., 2017). Above all, dealing with such unpredictability, as much as with the impairment of daily activities, makes this condition of particular relevance from a psychological point of view.

The caregivers of HAE patients experience the burden of such unpredictability of HAE attacks in daily life. Often the attacks can occur at school and parents have to leave their work schedules to reach their children. Moreover, some parents report difficulties in leaving the child as well as in letting the child/adolescent go far from them. The sense of unpredictability is made worse by the poor awareness of the disease and its medical protocols and prophylaxis within medical and social settings (Freda et al., 2016a; Savarese et al., 2017, 2018).

According to clinical reports, even with a lack of scientific evidence regarding cause-effect relationships, several factors are commonly associated to HAE attacks. The most commonly cited are physical as well as psychological triggers. The “psychological hypothesis” has a long tradition in the history of HAE since the first case identified in the medical literature was attributed to a nervous and psychosomatic cause (Ossler, 1888; Bannister, 1894; McDougall, 1989). Our previous pilot studies on HAE highlighted that clinicians should be aware that the common-sense hypothesis concerning physical and psychological triggers may lead to the development of unjustified and excessive fears of engaging in physical and social activities, as well as to an attitude of avoidance and denial toward negative emotions and the events that may potentially trigger them (Freda et al., 2016a; Savarese et al., 2018). It is therefore necessary to find effective ways to collect anamnestic information and to share suggestions and knowledge on these aspects of HAE within the health-care system.

Making reference to the existing literature in the field (Bibace and Walsh, 1980; Belsky et al., 1995; Charman and Chandiramani, 1995; Marvin and Pianta, 1996; Koopman et al., 2004; Bohanek et al., 2005; Fivush and Sales, 2006; Milshtein et al., 2009; Lecciso and Petrocchi, 2012; Eccleston et al., 2015) and to our pilot studies on hereditary angioedema in pediatrics (Freda et al., 2016a; Savarese et al., 2018), we identify three key domains of the disease experience that caregivers are called to face with, while constructing the sense of the disease:

A. The Interpretation of the Path of the Disease and Its Symptomatologic Variability

This domain refers to all those sensemaking processes, starting from the moment the first diagnosis is delivered (Good, 1994; Marvin and Pianta, 1996; Charon, 2008), that are an attempt to narratively deal with the variability of HAE symptoms. It refers to the specific way in which parents manage to integrate and connect medical information and their knowledge/beliefs, generating original and creative ways of interpreting and explaining the disease in their lives (Leventhal et al., 2001). The specificity of the disease, together with a number of contextual and subjective variables, imply a variable degree of medical literacy and understanding of the etiology and the factors triggering the symptoms, reducing or accentuating the uncertainty and chaos with which parents have to deal with in the sensemaking of the illness (Oppenheim et al., 2007).

B. The Dialogical Processes Related to the Disease in the Family

For young patients, the possibilities offered by dialogue represent a central issue of development, growth, and coming to terms with their illness (Campbell and Wales, 1970; Bibace and Walsh, 1980; Charman and Chandiramani, 1995; Koopman et al., 2004).

Dialogue on the illness within the family allows an opportunity to share medical information as well as to address many other issues: collecting information on the disease, providing a space for the child to share about the disease from an emotional perspective, and about talking about it with significant others. Within this domain, it is possible to observe how the various dialogical modalities of sharing information and knowledge between caregivers and children, and even brothers and sisters, can respond to specific functions (Sommantico et al., 2017). Each caregiver and family differs in terms of dialogical styles as well as for a greater or lesser ability to accompany their child in the construction of meaning of the path of illness (Clarke et al., 2005). There is no right way to define dialogic processes: the effectiveness of dialogical exchanges can be considered in terms of being attuned with a young patient, with her cognitive and affective needs, with her ability to understand, contain, and make use of various levels of information (Dicé et al., 2017, 2019).

C. The Management of the Disease in the Daily Life of the Family

Managing a chronic disease in everyday life is a central dimension of this experience (Williams, 1984). Within it, we find choices, actions, behaviors, attitudes, and strategies: all these agentive processes are manifestly of implicitly goal-oriented. In the early days of an illness, the urgency of managing the disease can precede the most basic understanding of the condition itself (Cohen, 1999).

Before gaining sufficient understanding, caregivers are called upon to administer therapies, take precautions, and modify everyday-life activities to follow the therapeutic indications they receive. At this time, the accumulation of direct experiences about the illness and the widening of its understanding can contribute to better strategies of dealing with it, switching from the mere execution of medical suggestions to integrated actions in daily life (Grande et al., 2014; Graffigna et al., 2017; Venezia et al., 2019).

These domains are separate but intertwined: the relation between interpretation, dialogue, and disease management has been explored in a number of publications (Walsh, 2003; Quattropani et al., 2018a,b; Martino et al., 2019a,b). Nonetheless, the literature is ambiguous when establishing which domain is the cause of the other domain’s characteristics. The confusion is mostly attributed to the role of sensemaking processes and the interpretation of the disease regarding management and coping strategies (Brandes and Mullan, 2014; Dempster et al., 2015). In the light of these last considerations, the aim of this article is to explore how caregivers narratively deal with the high variability and uncertainty of HAE in their daily life and identifying the link between their interpretation of the disease and the dialogic and agentive processes toward their children’s disease.

The theoretical background for our study relies on a narrative and semiotic conception of the mind which is grounded in a narrative, neurobiological, and semiotic literature (Gazzaniga and LeDoux, 1978; Polkinghorne, 1988; Bruner, 1990; Freda, 2008). In our view, the mind is conceived as a narrative semiotic system for the organization of the elements of experience, responding to the needs of continuity and coherence of the self (Lichtenberg, 1988; Proulx and Inzlicht, 2012; Lichtenberg et al., 2017). Semiotic studies (Peirce, 1935; Eco, 1976; Greimas et al., 1982; Sebeok and Danesi, 2000) have provided significant contributions to the psychological sciences by conceptualizing the mind as a dynamic of sensemaking within a semiotic flow.

The sense of experience is therefore constructed with a dynamic articulation of signs between the subject, the symbolic environments, and other social actors (De Luca Picione et al., 2017). While connecting and organizing, the narrative function of the mind “interprets” (Gazzaniga and LeDoux, 1978; Barrett, 2017) the experience on the basis of previous experiences, making inferences on the expected relations between future ones (Bruner, 1990; Valsiner, 2007, 2014).

With reference to our research topic, narration is a widely acknowledged device of elaborating one’s own illness experience, to reflect on it and to share subjective aspects (Bruner, 1990; Good, 1994; Pennebaker and Seagal, 1999; Charon, 2008; Park, 2010; Fioretti and Smorti, 2016; Freda et al., 2016b; De Luca Picione and Valsiner, 2017). Facing the experience of illness and its diagnosis, the narration responds to the need to reorganize, to restore a form, and to reposition one’s own identity (Martino et al., 2019a,b). Simultaneously, the constructive and interpretative functions of narration are challenged by the character of novelty and violation of the canonical, and the uncertainty is carried out by the experience of illness itself (Bruner, 1990). In the specific case of HAE, the illness narration is called to deal with a high intra- and inter-individual variability and unpredictability. The chronicity of the condition, moreover, forces patients to face the continuous repetition of critical events of the disease and its changes over time. The sensemaking of the disease and the possibility of relying on the narrative understanding based on previous experiences are therefore continually challenged by the variability of the disease and the novel forms it shows within different developmental stages and life events (Bury, 1982).

## Materials and Methods

The study presents a qualitatively driven mixed-method research design (Morse and Cheek, 2014) that relies on: (1) a qualitative narrative semiotic analysis (Bruner, 1990; Valsiner, 2007; Freda, 2008; Salvatore, 2015; De Luca Picione et al., 2017) of interviews with mothers of young HAE patients; (2) the SPSS (Version 23) TwoStep Cluster algorithm (described below) for categorical data to identify the profiles of the maternal sensemaking processes of the disease between the selected domains of the disease experience, empirically rather than theoretically. In order to verify the effectiveness of the categories detected with a qualitative approach, we decided to define the profiles using a statistical-computational approach. Relying on the theoretical background made explicit in the last section, we executed a semiotic narrative analysis of the maternal sensemaking processes of their children’s disease, where with “sensemaking processes,” we refer to the logic of the narrative construction of links between the elements of the experience, as well as to the general purpose and objectives to which the narration, in its agentive function, responds (Peirce, 1935; Baldwin, 2009; Proulx and Inzlicht, 2012; De Luca Picione et al., 2019). Therefore, analytical attention was not only focused on the semantic contents of the narration but also on the characteristics of the links that orient the connection between the elements of the experience within each domain: (1) the connection between events, for the interpretation of disease variability domain, (2) the connection between self and other, for the dialogue domain, and (3) the connection between sensemaking and actions, for the disease management domain. This analytical level is what we refer to as “sensemaking modalities” (SM) within the coding grid described below. The adopted semiotic perspective is devoted to grasping the “how” (namely, the process in contextual and relational terms) rather than the “what” (i.e., isolated and specific objects). In some cases, we also relied on the analysis of some linguistic markers aiming to grasp these characteristics of the narrative links between the elements of the experience.

### Participants

This research was conducted within the framework of a multi-centric study with the involvement of all Italian referral centers for HAE. In this article, we refer to n. 28 interviews with mothers of children aged 8–14 years, who received the diagnosis at least 2 years before recruitment. In the overall research design, the collection of both fathers’ and mothers’ narratives was envisaged, but only few fathers (n. 5) agreed to participate in the research meetings. In this paper, we therefore decided to analyze only the mothers’ narrative aiming at comparing a more homogeneous corpus. The mean age of mothers was 41.3 (SD ± 5.7), and the level of education was average for 72% and poor for 28%. The marital status at the time of the research was married for 87% of the sample. The mean age of the children was 11.4 (±4.6). The rejection percentage was 15%, mostly due to logistics (e.g., living far away from the meeting locations, difficulties with working hours, etc.). Participants signed an informed consent to participate in the research and a privacy statement on the most recent legislation on the treatment of personal data (EU GDPR 2016/679).

### Data Collection

A team of two trained clinical psychologists and psychotherapists executed face-to-face individual interviews with the mothers. The average length of the interviews was 20 min. Each interview was audio-recorded and transcribed verbatim by a trained clinical psychologist according to the APA rules for privacy and respect for the participants.

### Research Tools

#### The *ad hoc* Narrative Interview

We developed an *ad hoc* narrative semi-structured interview aimed at exploring the sensemaking processes related to the three key domains of the disease experience of chronicity introduced in the paragraph above: (1) interpretation, (2) dialogical processes, and (3) management. Each question of the interview was directed to eliciting the processes for the understanding and the organization of the disease experience (McIntosh and Morse, 2015).

The interview lets the narrator recount her experience and organize it, yet some questions are asked by the interviewer, for example, soliciting both a diachronic perspective – about the changes and transformations in the time – and an episodic perspective (i.e., “Could you tell me one of the most critical events within the last 6 months connected to HAE?”). The current version of the interview has n. 11 questions (see Table 1). It was drawn up during the research process after an initial prototype of the interview was submitted to six preliminary subjects.

**Table 1:** The semi-structured interview of the parental sense of grip on the disease of their children.

Interview on parental sense of grip on the disease	
1	When and how did you discover that your child suffers from a medical condition?
2	When did you realize that he was affected by (*name of the disease*)? How did you feel?
3	In your family experience, are the symptoms associated with anything in particular? (if they refer to emotions, ask: what do you mean by emotion/stress?)
4	How are the (*name of the disease*) symptoms these days?
5	What do you do to take care of (*name of the disease*) in your daily life?
6	In there something or someone that you see as a support in dealing with the disease?
7	How do you talk about (*name of the disease*) in your family? Which words do you use to define it?
8	Has the way you talked about (*name of the disease*) changed over the years?
9	In your opinion, what does your child think of it? Does she/he asks questions? According to you, what does your child know about the disease?
10	Tell me about a salient symptomatic episode/the one that was most significant and recent for you (within the last six months or, if there has not been one, within the last year)
11	In this situation, in your opinion, things would have gone differently if...
…	Do you want to add something that we did not ask?

As a case in point, we report some of the questions of the interview: for the exploration of the first domain of disease “interpretation,” one of the questions was as follows:

“How are the HAE symptoms these days”; for the “dialogical processes” domain, one of the questions was as follows: “How do you talk about HAE in your family?…. In your opinion, what does your child know about the disease?”; for the “management” of the disease domain, one of the questions was as follows: “what do you do to take care of HAE in your daily life?”

### Analyses

The qualitative analysis of the narrative corpus was carried out by a group of three trained researchers with expertise in qualitative narrative analysis, alternating independent work with group work. In the cases of discordant classifications, the researchers worked together until they reached a shared and unanimous judgment. In line with our theoretical background, we started to approach the narrative corpus with the following general research questions:

1. How do mothers narratively deal with the variability and unpredictability of HAE in everyday life?
2. Which sensemaking processes are implemented while facing the different issues of the chronicity in HAE?

The analysis was therefore articulated in four main stages (Kawulich, 2004):

#### Labeling

In the first stage, we read each interview to understand the modalities of the sensemaking processes for each of the three domains. We then attributed a label to each sensemaking process for each domain within each interview. Three independent researchers carried out this stage of the analysis.

#### Summarizing and Categorizing

In the second stage, we carried out an analysis of all the interviews with the purpose of comparing these labels within each domain in order to summarize and categorize them within a limited number of modalities. As specified above, with the terms “sensemaking modality” (SM), we refer to the analytical level aimed at grasping the links that orient the connection between the elements of the experience within each domain: (A) the connection between events, for the disease variability interpretation domain, (B) the connection between self and other, for the dialogue domain, and (C) the connection among sensemaking and actions, for the disease management domain. These modalities respond to the criteria of exhaustiveness and mutual exclusion. In this stage, the three researchers worked together to reach a consensus on each modality identified.

#### Clusterization

Subsequently, according to the principles of qualitatively driven research (Guest et al., 2001; Morse and Cheek, 2014), which calls for the integration between qualitative and quantitative elaboration of narrative data, the TwoStep cluster analysis procedure for categorical data was executed. This procedure identifies the optimal number of clusters and the best solution among many potentially logical clusters, minimizing the Bayesian information criterion (BIC). The number of clusters can be also fixed by the users, so that if the computed solution is not satisfactory a different partition can be identified. Finally, the interpretation of profiles takes into account the distribution of each disease sensemaking domain across clusters, following an interpretive criterion to assign labels to them.

#### Creating a Conceptual Framework

Lastly, relying on an abductive logic (Salvatore, 2015) to conceptualize and model a psychological process, we propose the concept of *Sense of Grip of the Disease* to discuss the key elements of our findings.

## Results

### Results From the Semiotic Narrative Analysis of the Interviews: The Sensemaking Modalities for Each Domain of the Disease Experience

We discuss the sensemaking modalities (SM) identified for each domain of the disease experience.

A. Disease variability interpretation domain

We identified three SM for the articulation of this domain, referred to the processes of narrative sensemaking of disease origin, the determinants, and symptom triggers: (1) “closed,” (2) “hypothetical,” and (3) “confused” (see Table 2).

**Table 2:** Domain of the disease experience (A): the interpretation of the path of the disease and its variability. Sensemaking modalities (SM) and representativeness.

Domain of the disease experience	Sensemaking modality (SM)		Representativeness
(A) Interpretation of the disease and its variability	1	Closed	12 (43%)
	2	Hypothetical	11 (39%)
	3	Confused	5 (18%)

1. “Closed” modality (43%). It refers to narrative sensemaking processes that express the construction of closed and permanent causal links. The narrative is characterized by the presence (or absence) of causal relations between specific triggers and symptoms of HAE. Within these narratives, we detected specific linguistic indicators (Bongelli and Zuczkowski, 2008) that refer to certainty (e.g., “certainly,” “always,” “surely,” “no doubt,” etc.) and verb forms conjugated in the simple present (e.g., “it happens…”) without any sense of hypothesis. In such narratives, it is common to find a massive use of negations: they serve to support the unmistakable veracity of the causal link identified. This attitude informs an epistemic stance of the narrator as “sure” of her own statements on the disease.

Mother: “… attacks always occur after a fall of after he hurts himself accidentally. Stress makes him swell too…” (Int. n. 48, p. 3, lines 53–59).

Mother: “…My son has never swelled due to stress...and besides, which kind of stress should a 4 years old child experience” (Int. n. 9, p. 2, lines 45–46).

2. “Hypothetical” modality (39%). We refer to this as detecting sensemaking processes marked by an openness to possibility, probability, and doubt. The sensemaking process is expressed as a pronounced sensitivity to contextual differences and as an attitude to grasp the changes in the disease manifestations and tolerate uncertainty. This is expressed in linguistic markers of probability, including the verb “to believe,” “to guess,” “it seems that,” and “to suppose,” adverbs of doubt such as “maybe,” “sometimes,” “once,” and the conjunction “if”…

Mother: “…attacks don’t always occur for the same reason, I try to ask my son what happened before the attack, if he bumped into something, if he was nervous …. It seems that when he has the flu it happens more frequently, we made this association, that’s probably how it goes…” (Int. n. 3, p. 4, lines 13–16).

3. “Confused” modality (18%). This refers to the sensemaking processes based on the impossibility of finding clear causal links. The consequent confusion takes two different forms: the total absence of causal links or the hypertrophy and inconsistency of these links. In the former, the linguistic markers focus on absence and impossibility: “we don’t know,” “uhm,” while the latter form focuses on indicators of summation such as the adverbs “too” and “moreover,” associated to assertions like “yes,” “that’s how it goes…”

(Example on the impossibility of finding clear causal link)

Mother: “We don’t know when and how the attack will come, when it has to be it comes…”(Int. n. 6, p. 4, line 65).

(Example on the hypertrophy of the links)

Mother: “F. swells when he’s nervous due to a school test, or when he plays football…once it even happened while eating mussels when we were on a trip… it always happens when we are on a trip since being apart is a quite stressful experience for him…even if he enjoys traveling with me a lot !” (Int. n. 13, p. 3, lines 32-36).

B. Dialogical processes domain

We detected five SM for this domain referred to in the dialogical processes of the diverse aspects of the disease experience between mothers and children: (1) “pragmatic,” (2) “alarmistic,” (3) “neutralizing,” (4) “delegating,” and (5) “silent” (see Table 3).

**Table 3:** Domain of the disease experience (B): the dialogical processes related to the disease in the family. Sensemaking modalities (SM) and representativeness.

Domain of the disease experience	Sensemaking modality (SM)		Representativeness
(B) Dialogical processes	1	Pragmatic	7 (25%)
	2	Alarmistic	7 (25%)
	3	Neutralizing	7 (25%)
	4	Delegating	4 (14%)
	5	Silent	3 (11%)

1. “Pragmatic” modality (25%). This is expressed in communicative exchanges between parents and children built on the recognition of the child’s needs for knowledge and his/her capacities of comprehension in relation to his/her development stage. This modality of sensemaking is characterized by the ability to attune with the developmental affective and cognitive needs of children. An additional indicator of this modality is a diachronic format of the narrative in which a transformation in the dialogical exchanges between parents and children is present, as in the following narrative excerpt:

Mother: “…when he was younger we talked to him in a different way because we were afraid of making him feel “different” … when he asks me something I try to give him a complete/satisfactory answer because I believe that he, I don’t know if he asks himself anything on the future…but we have attempted to talk about it… so when he talked about joining the army we said “there may be some physical tests and you need to be 100% ok …” (Int. n. 25, p. 5, 189-198).

2. “Alarmistic” modality (25%). The goal of such dialogical exchanges is to warn children of what mothers interpret as the danger that HAE symptoms could arise according to their own narrative normative system. This reflects an exasperation of the potential risks for the disease onset in the implicit attempt to control mothers’ own anxieties. In such cases, the other’s perspective (namely, the child’s) is not effectively acknowledged. Talking about the disease is not addressed to the child but to the mother, to self-comfort themselves by frightening their children to control them.

Mother: “I have always told her, since I always talk to her, that (in case of her throat swelling) she is in danger of asphyxiation. And what could I do without her??? I always talk to her…” (Int. n. 15, p. 2, lines 22–24).

3. “Neutralizing” modality (25%). We identified this modality to classify cases where dialogues on the disease are aimed at minimizing it through overly optimistic tones. The sensemaking processes are saturated by positive and encouraging terms, at the cost, though, of excluding any space to share any negative emotion and feeling experienced by the child. Typical statements of such modality are “*she’s fine*” “*he’s not ill at all*,” “*he can do everything great!*” Classic statements are those in which a comparison with another more severe condition is made, e.g., “*I always tell him there are worse problems than HAE...*”

Mother: “…when she swells she starts crying “why does it happen to me!!! Everything happens to me!!! And suddenly I say “don’t worry, everything passes, this problem is better than others…” (Int n. 3, p.1, lines 20–22).

Mother: “My son is not worried at all because he has nothing!!”. (Int. n. 8, p. 7, lines 181–182).

4. “Delegating” modality (14%). This modality is designed to capture those narratives in which mothers prefer not to explicitly talk about HAE, with the explanation that somebody else takes care of it (medical staff, the other parent who is affected by HAE as well). In these cases, mothers seem to rely on other people and avoid talking about HAE except for practical therapeutic matters. Furthermore, in this case too, the narrative seems to respond to parental needs of controlling their own worries and difficulties in facing their children’s HAE.

Mother: “I didn’t tell him anything, the father who suffers from the same disease did it!”(Int. n. 18, p. 7, lines 694–698).

5. “Silent” modality (11%). We refer to this sensemaking processes in those narratives in which there is a complete absence of communication on issues related to the HAE experience. Parents avoid talking about HAE for various reasons: they do not feel like it or deliberately choose not to do so to overprotect the child from what is signified as a negative issue in the relation with his/her child.

Similar to the previous three modalities, in this case, the sensemaking comes into play to quell/sedate the unbearable emotions of parents.

Mother: “… no, I prefer not to talk about it …I don’t want to make him feel bad…he already has to live with all this…” (Int. n. 20, p. 3, lines 74–76).

Mother: “At home we try not to talk about it trying not to give him a bad time …” (Int. n. 4, p. 4, line 54).

C. Disease management domain

For the management of children’s HAE domain in daily life, we developed three SM: (1) “limiting-avoidant,” (2) “flexible,” and (3) “executive” (see Table 4).

**Table 4:** Domain of the disease experience (C): the management of the disease in the daily life of the family. Sensemaking modalities (SM) and representativeness.

Domain of the disease experience	Sensemaking modality (SM)		Representativeness
(C) Management	1	Limiting-avoidant	2 (7%)
	2	Flexible	17(61%)
	3	Executive	9 (32%)

1. “Limiting-avoidant” modality (7%). This modality is detected in those narratives in which a process of limitation or avoidance toward daily social and leisure activities is referred to as the strategy of choice for dealing with HAE. Such limitations seem excessive when faced with the set of medical provisions suggested by clinicians. The narratives in such cases underlie a risk of inhibiting the basic experience of socialization that is usual for children of that age.

Mother: “We retired him from karate, we don’t let him practice any sport and he can’t go far from home…we keep him under a glass bell” (Int. n. 15, p. 3, lines 68–69).

2. “Flexible” modality (61%). We attribute this classification to those narratives in which there is no unique strategy to deal with the disease. Parents rather refer to an ongoing negotiating process between the desires and needs of the child and the limitations imposed by the disease. This results in the gradual development of the child’s autonomy and responsibility for his/her own health management. In these cases, the narrative expresses to the best of his/her potential the desire to foster the child’s decision-making autonomy toward the disease.

Mother: “I try not to limit her freedom. When she asks me to practice a sport we try to find the one that best fits for her, for example she wanted to do modern dance and I suggested she opt for a musical because it's still fun, but requires less effort…” (Int. n. 10, p. 7, lines 191–193).

Mother: “We also had to inform the parents of the other guys, last summer he went camping so we told his friend’s mother of: “…you have to keep it (the drug) in the refrigerator, hoping that there will be no need to use it! And anyway you can call us!” (Int. n. 17, p. 5, lines 3–5).

3. “Executive” modality (32%). This modality is attributed to those narratives in which the main management strategy is the absolute adhesion to the medical advice and prescriptions. The *agentive* function of the narrative does not leave space to any negotiation between these prescriptions and subjective experiential capital. Classic for this modality are the narratives in which, at the question regarding actions undertaken to deal with the disease, parents merely enumerate the therapeutic prescriptions of the physician to the letter. We are dealing with relationships based on compliance and therapeutic adherence.

Mother: “When the attack occurs we administer the drug. Doctor X said that a quicker administration is always better” (Int. n. 24, p. 6, lines 13–14).

### Analysis of the Trajectories Between the Domains: The Profiles

We performed the TwoStep Cluster Analysis procedure for categorical data on the codebook derived from the transformation of the qualitative coding process of the SM into nominal variables. The analysis generated three clusters (for which the silhouette measure – fair – stands on a sufficient level of cohesion and separation), which were interpreted in terms of recursive patterns by which the SM are organized in the domains of the disease experience (see Table 5). We also explored the two- and four-cluster solutions, but the three clusters optimal solution was the most satisfactory from an interpretive point of view.

**Table 5:** Summary of the profiles interpreted by the TwoStep Cluster analysis on n. 28 interviews to mothers of children with HAE, with the frequencies of the SM within each domain of the disease experience.

	Cluster n.1	Cluster n.2	Cluster n.3
(A) Interpretation	0 Hypothetical 6 Closed 4 Confused	4 Closed 3 Hypothetical 0 Confused	2 Closed 8 Hypothetical 1 Confused
(B) Dialogical processes	1 Pragmatic 3 Alarmist 2 Neutralizing 2 Delegating 2 Silent	0 Pragmatic 0 Alarmist 5 Neutralizing 2 Delegating 0 Silent	6 Pragmatic 4 Alarmist 0 Neutralizing 0 Delegating 1 Silent
(C) Management	1 Limiting 9 Executive 0 Flexible	1 Limiting 0 Executive 6 Flexible	0 Limiting 0 Executive 10 Flexible
Profile	Adempitive	Reactive	Dynamic

The first profile, defined as *adempitive*, is mainly described by executive management and the absence of interpretative processes with a hypothetical modality. This may suggest that the management processes, based on mere execution, are the result of interpretative processes that leave no space to the variability of the experience and express the inability to tolerate uncertainty, such that the only possible regulation of the disease is the execution of directives that come from outside (i.e., medical prescriptions). Dialogue processes are also prevalent in ways that indicate difficulty in acknowledging the child’s knowledge and health needs. It also seems difficult for these mothers to take responsibility for speaking about negative emotions, or communicating with their children in general.

The second profile, defined as *reactive*, is mostly represented by neutralizing dialogic processes and flexible coping. The attempts to neutralize the negative experiences connected to the illness can support strategies for the flexible and effective management of the disease in everyday life. In dynamic terms, this configuration corresponds to an adaptive psychological mechanism of defense based on management that focuses on facilitating daily actions to face and integrate the disease, though it is less reflective. For these reasons, it has been labeled “reactive.”

The third profile, defined *dynamic*, is identified in a flexible management (all of the cases that compose it) in a hypothetical interpretation and in dialogic processes that are mainly pragmatic. Flexible adjustments are associated with normative processes in which one is able to tolerate uncertainty and remain open to reading the variability of experience. This also corresponds to a greater ability to recognize the child’s requirements for specific and different needs.

## Discussion

Analysis of the results shows that the significant recourse (43%) to interpretative processes for the symptoms and the variability of the disease, that we have defined as “closed” and is characterized by the definitive identification of triggers of angioedema attacks, was mainly associated with “executive” or “limiting-avoidant” management processes, as the *adempitive* profile shows. In contrast, in cases where the interpretation was based on the formulation of “open” hypotheses (to be tested for the variability of situations), there was an association mainly with “pragmatic” dialogic processes based on the recognition of the child’s needs for knowledge and on “flexible” management strategies of contingent situations. This is the case of the *dynamic* profile. These results confirm what was already highlighted in our pilot study on hereditary angioedema in pediatrics (Freda et al., 2016a; Savarese et al., 2018) namely that caregivers’ detection of triggering causes (both physical and psychological) leads to limitations of children’s activities or to following general therapeutic indications slavishly, without these being adjusted to one’s own unique experiential baggage. This massive recourse to “closed” SM in the experience of hereditary angioedema can be interpreted as the other side of the wide uncertainty related to the triggers of HAE attacks and the high intra and inter individual variability of their occurrence. That is to say that the sensemaking reaction to high uncertainty is its opposite process: a sense of dogmatic certainty (Mishel, 1999). Considering the specificity of HAE, it is plausible that this use of the “closed” SM satisfies the need for stability and narrative continuity.

Furthermore, the results seem to suggest that, in the more virtuous sensemaking processes, the best answer to the uncertainty and the extreme variability of HAE symptoms corresponds to the ability to tolerate these circumstances and to be open to reading the contextual variability of HAE and the needs of the child.

This ability seems to lead to dealing with the elements of the intrinsic variability of HAE more effectively, going beyond the mere implementation of standardized management strategies and protocols.

### The Construction of a General Conceptual Framework

**Figure 1:**
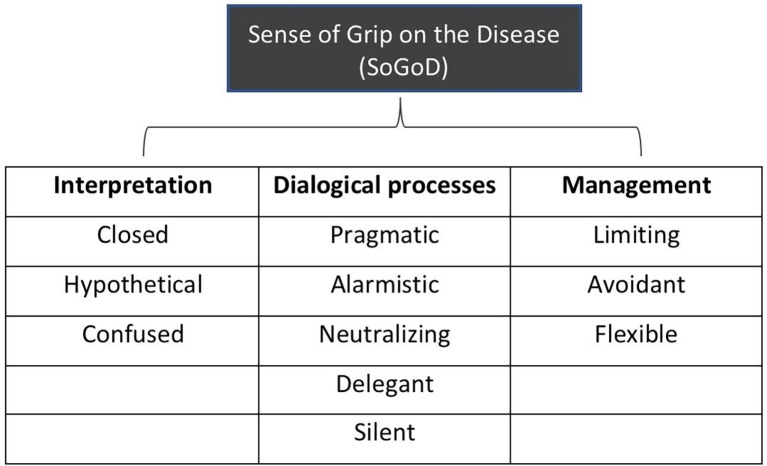
Domains of the sense of grip on the chronic disease (SoGoD) and their sensemaking modalities (SM) from the semiotic narrative analysis.

In our opinion, the profiles that have emerged in our analyses can be discussed making reference to a comprehensive tension to generate sense resources that can be used to negotiate the relation between the needs and constraints imposed by the disease and the contexts of everyday life (De Luca Picione et al., 2017, 2018). According to our semiotic perspective, this tension can be approached with the notion that we define as Sense of Grip on the Disease (SoGoD). The key words of this definition are the terms “grip” and “sense”: the first term refers to mastery and development of competence in a given domain, whereas the second term highlights the narrative constructive matrix of the process. It starts from the constructive and interpretative matrix of the mind (Freda, 2008; De Luca Picione et al., 2017), where the sense is constructed. Namely, what the person will mean as effective/appropriate or not for the integration of the disease in everyday life. The quality and the health outcomes of the caregivers’ Sense of Grip on their children’s disease are determined by the configurations that the different SM assume between the domains of the disease experience. The sensemaking processes within these domains, therefore, may be conceived as the essential components of the Sense of Grip itself, as we represent in Figure 1.

The sensemaking framework for caregivers’ interpretation of the disease and its variability contributes to orienting the dialogic exchanges and the daily management practices of the disease. Simultaneously, actions shape the contexts and intervene to modify or crystallize the dialogical and agentive processes. However, this is not a process that is exhausted at a specific moment, but is an ongoing sensemaking process in continuous evolution depending on variables such as the time elapsed from the diagnosis, emotional and cognitive processing, knowledge of the pathology, enabling factors of the health context, and the resilience resources available in a given context.

These results highlight the importance of sensemaking processes which, instead of relying on a coherent and closed interpretation of the disease, are characterized by a degree of tolerance for the uncertainty associated to hereditary angioedema. This tolerance is an indicator of caregivers’ competence in dealing with the variability of the HAE experience and adopting more flexible strategies for its management if the daily life.

In line with these reflections, we therefore believe that the nonhomogeneity within the literature on the relation between interpretive processes of the disease and its physical and psychological management processes should be attributed to the wide multi-causality and nonlinearity of the spectrum of adjustment processes to chronic disease (De Ridder et al., 2008; Moss-Morris, 2013). These processes, to which we refer as Sense of Grip on the Disease, are the product of complex interactions between subjective experiences, social and contextual resources, inputs by the medical settings, and the characteristics of the disease. Therefore, as the narrative analysis shows, the effectiveness of the processes is not detectable in one sensemaking strategy or another, but on the integration between competences of differentiating and making sense in a flexible and situated manner. This awareness plays a key-role in designing and orienting clinical psychological intervention in the field of chronic diseases. Psychological interventions should play a function of mediation between the healthcare system and the person, aiming to foster a process of elaboration and transformation of the standardized protocols suggested by medicine into narrative norms and actions suitable for the subjectivity of the patient and its caregiving system (De Luca Picione et al., 2017; Venuleo et al., 2018).

### Limits and Future Developments

Starting from this study, we aim in the near future to extend the evaluation of the modalities identified to understand the experience of HAE, to other chronic diseases in the pediatric age group to verify the validity and generalization of the identified psychological processes. Furthermore, we intend to evaluate the correlation between the severity of the disease, the time from diagnosis, and sense of grip profiles in HAE.

This assessment also includes a diachronic perspective that allows us to grasp any differences in the sense of grip in various periods of the experience of the disease. Moreover, a limitation of this study is that we could not analyze and compare the fathers’ narratives due to their scarce participation, since it could help to gain a wider view on the sensemaking of the familial caregiving system.

The evaluation should also be extended in the direction of identifying any additional predictors or intervening variables associated with the different configurations of the sense of grip, such as the specificity of the pathology under examination. The evaluation of the maternal sense of grip may prove to be a resource that allows the following: (1) to highlight the domains of risk and resources of the maternal sensemaking processes of a specific condition and (2) to develop *ad hoc* interventions using an integrated setting and flexibility between medicine and psychology. Lastly, we foresee the construction of a structured coding system that may represent a useful tool for clinicians interested in working with caregivers of children with chronic diseases.

## Data Availability Statement

The datasets generated for this study are available on request to the corresponding author.

## Ethics Statement

This study was executed under the approval of the Ethical Committee of the University Hospital Federico II of Naples (protocol n. 118/16). All procedures performed in this study were in accordance with the ethical standards of the study coordinating center and with the 1964 Helsinki Declaration and its later amendments or comparable ethical standards. Prior to study participation, participants signed an informed consent document.

## Author Contributions

MF helped in substantial contributions to the conception and design of the work, revising it critically for important intellectual content. LS helped in substantial contributions to the conception or design of the work, to the acquisition, analysis and interpretation of data, and drafting the work. PD helped in substantial contribution to the acquisition, analysis, and interpretation of data. RD helped in substantial contributions to the conception and design of the work, drafting the work, and revising it critically for important intellectual content.

### Conflict of Interest

The authors declare that the research was conducted in the absence of any commercial or financial relationships that could be construed as a potential conflict of interest.
